# Host Species and Geography Differentiate Honeybee Gut Bacterial Communities by Changing the Relative Contribution of Community Assembly Processes

**DOI:** 10.1128/mBio.00751-21

**Published:** 2021-06-01

**Authors:** Yuan Ge, Zhongwang Jing, Qingyun Diao, Ji-Zheng He, Yong-Jun Liu

**Affiliations:** a State Key Laboratory of Urban and Regional Ecology, Research Center for Eco-Environmental Sciences, Chinese Academy of Sciences, Beijing, China; b Department of Honeybee Protection and Biosafety, Institute of Apicultural Research, Chinese Academy of Agricultural Sciences, Beijing, China; c University of Chinese Academy of Sciences, Beijing, China; d Laboratory for Humid Subtropical Eco-geographical Processes of the Ministry of Education, Fujian Normal University, Fuzhou, China; CEH-Oxford

**Keywords:** honeybee, gut microbiota, bacterial community, host, geography, stochasticity, neutrality, selection, ecological processes

## Abstract

Honeybee gut microbiota modulates the health and fitness of honeybees, the ecologically and economically important pollinators and honey producers. However, which processes drive the assembly and shift of honeybee gut microbiota remains unknown. To explore the patterns of honeybee gut bacterial communities across host species and geographical sites and the relative contribution of different processes (i.e., homogeneous selection, variable selection, homogeneous dispersal, dispersal limitation, and an undominated process) in driving the patterns, two honeybee species (Apis cerana and Apis mellifera) were sampled from five geographically distant sites along a latitudinal gradient, followed by gut bacterial 16S rRNA gene sequencing. The gut bacterial communities differed significantly between *A. cerana* and *A. mellifera*, which was driven by the interhost dispersal limitation associated with the long-term coevolution between hosts and their prokaryotic symbionts. *A. mellifera* harbored more diverse but less varied gut bacterial communities than *A. cerana* due to the dominant role of homogeneous selection in converging *A. mellifera* intestinal communities. For each honeybee species, the gut bacterial communities differed across geographical sites, with individuals from lower latitudes harboring higher diversity; also, there was significant decay of gut community similarity against geographic distance. The geographical variation of honeybee gut bacterial communities was mainly driven by an undominated process (e.g., stochastic drift) rather than variable selection or dispersal limitation. This study elucidates that variations in host and geography alter the relative contribution of different processes in assembling honeybee gut microbiota and, thus, provides insights into the mechanisms underlying honeybee gut microbial shifts across evolutionary time.

## INTRODUCTION

Honeybees are important insects that provide crucial pollination services for terrestrial ecosystems and valuable apiarian products for humans (1). However, the populations of most honeybee species have declined worldwide over the past decade, probably due to augmented stressors such as toxins, pathogens, and poor nutrition, which might be tempered by the gut bacterial communities through their roles in detoxifying toxins, resisting pathogen infection, and prompting nutrient assimilation (2–6). Understanding the processes that govern honeybee gut bacterial communities is imperative for better managing gut microbiota to improve honeybee health and fitness, particularly in the context of increased environmental disturbances and global changes.

After years of research, we now know that honeybee gut microbiota is predominated by several core species clusters with distinct niche occupancy and functional complementarity, including those associated with host nutrition uptake (e.g., the genera *Lactobacillus*, *Gilliamella*, *Snodgrassella*, *Bifidobacterium*, and *Commensalibacter*), health promotion (e.g., the genera *Gilliamella* and *Bifidobacterium*), and pathogen defense (e.g., the genera *Lactobacillus*, *Gilliamella*, *Snodgrassella*, and *Bifidobacterium*) (2, 4, 6, 7). Determinants of honeybee gut bacterial communities include host attributes (e.g., species, age, and caste), external environment (e.g., diets, antibiotics, contaminants, and invasive colonizers from environmental sources), and probably geographical locations (2, 8–11). These studies have initiated new insights into the composition of honeybee gut bacterial communities and the factors that influence them. However, it remains unknown which processes drive the assembly and shift of honeybee gut bacterial communities.

**FIG 1:**
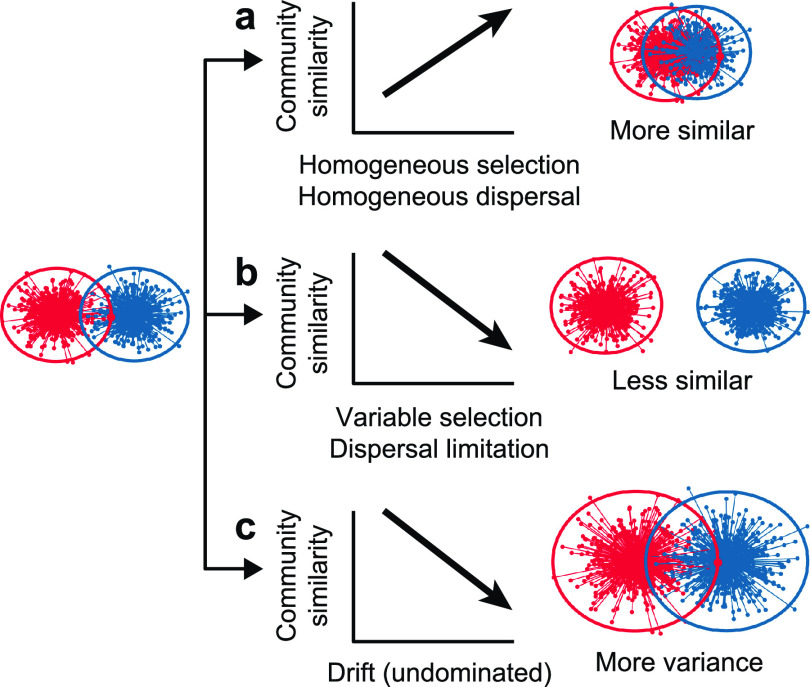
Dynamics of community patterns under the force of different processes. (a) Homogeneous selection and homogeneous dispersal cause communities to converge. (b) Variable selection and dispersal limitation cause communities to diverge. (c) Stochastic drift (i.e., undominated process) disperses communities.

Selection, dispersal, and drift have been proposed to be the major processes that govern the assembly and shift of ecological communities (12–15). The niche theory emphasizes the deterministic forces of biotic and abiotic factors in sorting communities (12, 16, 17). Selection may cause communities to converge if they undergo similar environmental conditions (homogeneous selection; Fig. 1a) or diverge if they undergo distinct environmental conditions (variable selection; Fig. 1b). The neutral theory emphasizes the stochastic processes such as dispersal and drift (13, 16, 18, 19). Dispersal influences community assembly by regulating the movement of species across spaces and systems. Dispersal can also converge or diverge communities depending on the magnitude of dispersal, that is, high dispersal homogenizes communities through the frequent species exchange between communities (homogeneous dispersal; Fig. 1a), while restricted dispersal differentiates communities (dispersal limitation; Fig. 1b). Ecological drift results in stochastic population fluctuation of component species in communities by chance birth and death events and, thus, generally disperses communities (Fig. 1c). For honeybee gut bacterial communities, selection, dispersal, and drift may also be the underlying processes that concurrently determine community assembly and shift. The key issues are determining their relative quantitative importance and how their relative importance varies across biological and spatial scales.

To answer these questions, we deep sequenced the 16S rRNA genes of 100 gut samples that were associated with two honeybee species (Apis cerana and Apis mellifera) and five geographical sites along a latitudinal gradient (distance of up to 1,000 km). We then examined the patterns of honeybee gut bacterial communities across host species and geographical sites. Finally, we quantitatively deciphered the relative importance of selection, dispersal, and drift in governing the assembly of honeybee gut bacterial communities and explored the shifts of these processes across biological and spatial scales, e.g., within versus between host species and within versus between geographical sites. We hypothesized that host species and geography can differentiate honeybee gut bacterial communities by enhancing the relative contribution of specific processes that diverge communities, e.g., variable selection, dispersal limitation, or drift.

## RESULTS AND DISCUSSION

### Host species determines honeybee gut bacterial communities.

**FIG 2:**
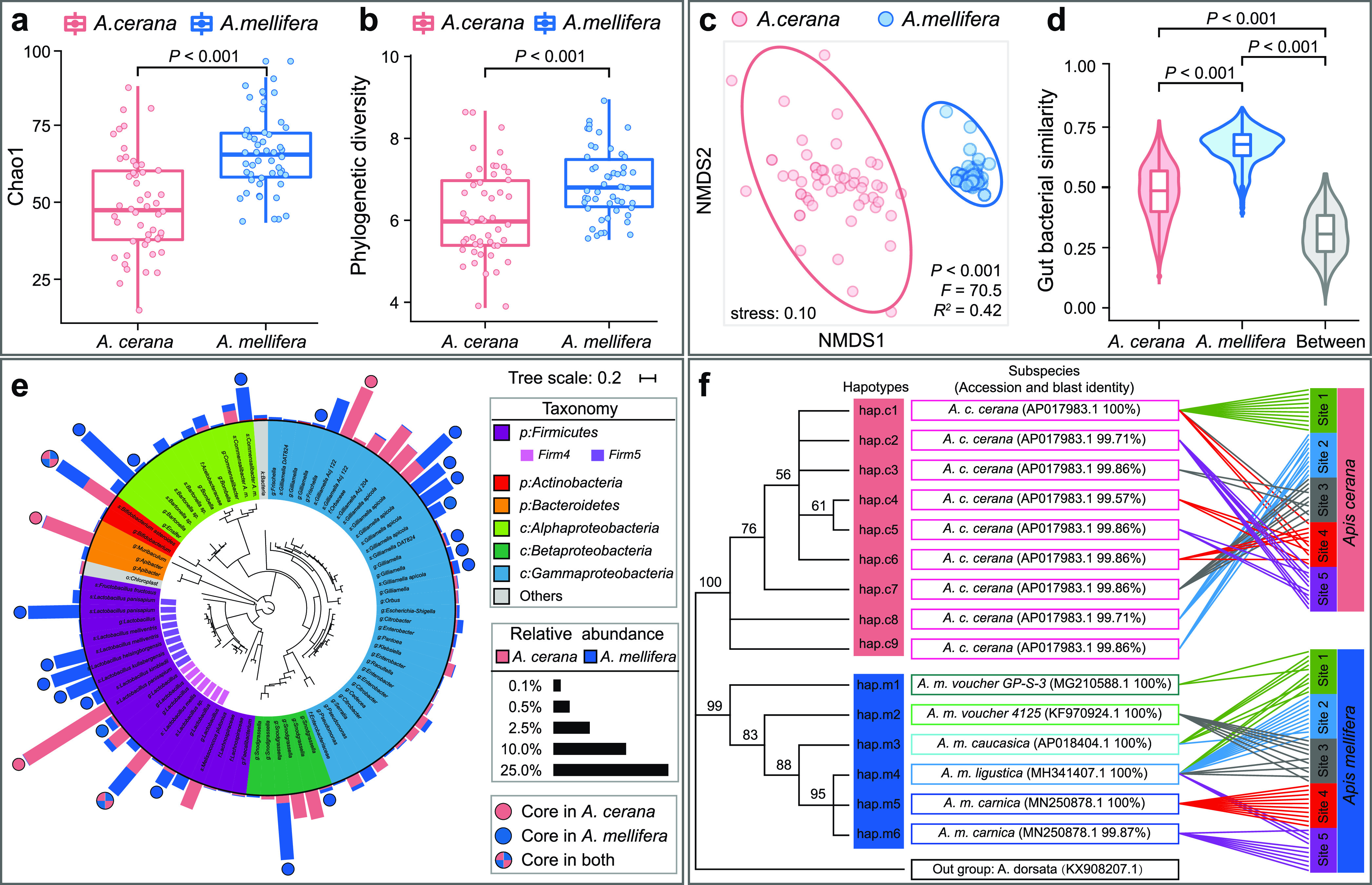
Distinct gut bacterial communities between *A. cerana* and *A. mellifera*. The Chao1 richness (a) and phylogenetic diversity (b) of *A. mellifera* gut bacterial communities were higher than those of *A. cerana*. (c) Nonmetric multidimensional scaling (NMDS) profiling illustrated distinct gut bacterial communities between *A. cerana* and *A. mellifera*. (d) Comparison of gut bacterial similarities within *A. cerana*, within *A. mellifera*, and between host species. (e) Phylogenetic tree of the 81 operational taxonomic units (OTUs) with a relative abundance of >0.1% in at least one sample, where the core OTUs within *A. cerana* and *A. mellifera* guts were labeled with red and blue circles, respectively. Bars showed the mean relative abundance of the OTUs. (f) The bootstrap consensus tree of the COI-COII haplotypes observed in this study and the correspondence to 100 honeybee samples. Branches with less than 50% bootstrap replicates were collapsed. Numbers on the edges of the tree represent the bootstrap values.

Despite the close phylogenetic relatedness between *A. cerana* and *A. mellifera* (20), the gut bacterial communities differed between these two honeybee species (Fig. 2). For example, the richness (Chao1) and phylogenetic diversity of *A. mellifera* gut bacterial communities were 34% and 13% higher than *A. cerana* (*P < *0.001 for both; Fig. 2a and b), manifesting the driving force of host species in determining honeybee gut bacterial diversity. The typically smaller body and colony size of *A. cerana* may partially explain their less diverse gut microbiota (21).

The overall intestinal community composition also differed between *A. cerana* and *A. mellifera*, as reflected by the host-associated community separation visualized in the nonmetric multidimensional scaling (NMDS) profile and the significance test based on permutational multivariate analysis of variance (PERMANOVA; *P < *0.001; Fig. 2c). The gut bacterial similarities of *A. mellifera* were 36% higher than those of *A. cerana* (*P < *0.001; Fig. 2d), indicating that the gut bacterial communities of *A. mellifera* were less varied across honeybee individuals than *A. cerana*. The gut bacterial similarities within the same host species (i.e., either *A. cerana* or *A. mellifera*) also were significantly higher than those between host species (*P < *0.001; Fig. 2d), further suggesting the role of host species in differentiating honeybee gut bacterial communities. These results indicate that honeybee gut bacterial communities have evolved intimate associations with their hosts through long-term coevolution; thus, different honeybee species possess characteristic gut microbiota (2, 21–23).

Besides the overall bacterial properties (e.g., diversity and community composition), the core OTUs (operational taxonomic units) also differed between host species (Fig. 2e). We defined the core OTUs as those observed at all of the five sampling sites (occurrence in ≥8 of 10 honeybees at each site). Among the 274 bacterial OTUs detected in this study, 5 and 22 OTUs were identified as the core OTUs of *A. cerana* and *A. mellifera*, respectively (Fig. 2e). These core OTUs accounted for 69.1% of *A. cerana* and 89.6% of *A. mellifera* gut bacterial abundances. The 5 core OTUs of *A. cerana* were assigned to the genera *Lactobacillus* (including 2 OTUs; with 1 Firm4 and 1 Firm5), *Bifidobacterium* (1), *Gilliamella* (1), and *Apibacter* (1); the 22 core OTUs of *A. mellifera* were assigned to the genera *Lactobacillus* (8; 3 Firm4 and 5 Firm5), *Gilliamella* (8), *Bartonella* (2), *Snodgrassella* (2), *Bifidobacterium* (1), and *Commensalibacter* (1). Among these core OTUs, 2 OTUs, assigned to Lactobacillus mellis (Firm4-1) and Bifidobacterium asteroides (Bifido-1), respectively, were shared by both host species. Interestingly, Ellegaard et al. also observed only two specific clusters (annotated to Firm4-1 and Bifido-1) that were shared between *A. cerana* and *A. mellifera* using the shotgun metagenomic method (9). We further searched the two shared core OTUs in our study against the same reference genomes used by Ellegaard et al. and found that they were from the same clusters observed by Ellegaard et al. (Bifido-1, with BLAST identities of 99.1 to 100%; Firm4-1, 99.3 to 99.8%) (9), which suggested that the resolution of 16S rRNA gene sequencing and OTUs (>97% identity) are adequate to characterize honeybee gut bacterial diversity.

Most of the identified core OTUs (e.g., *Lactobacillus*, *Gilliamella*, *Snodgrassella*, *Bifidobacterium*, and *Commensalibacter*) were reported to perform essential functions in nutrition uptake, health promotion, and pathogen defense for their hosts (2, 4, 7). The identification of core OTUs allows establishing a parsimonious list of preferred species that could be used in future community manipulation experiments for better understanding and managing honeybee gut microbiota to improve host health and fitness (3, 4, 7, 24).

Despite the dominant role of honeybee species, the genetic variance between haplotypes of the same honeybee species had no influence on the gut bacterial communities. We sequenced the mitochondrial COI-COII region (691 to 1,061 bp) of the 100 honeybee individuals to assess the relationship between host genetic diversification and gut bacterial community variation. To validate the use of the COI-COII region to infer honeybee genetic diversity at the species or subspecies level, we analyzed all the available whole genomic mitochondrion sequences of both *A. cerana* and *A. mellifera* accessed from GenBank, and the result showed that the genetic diversification in the COI-COII region can perfectly reflect the honeybee whole genomic mitochondrion genetic diversification (assessed by Mantel correlation [see Fig. S1 in the supplemental material]; *R *= 0.99 at species and subspecies levels; *R *= 0.99 at the *A. mellifera* subspecies level; *R *= 0.93, *A. cerana* at the subspecies level; *R *= 0.68 between different *Apis* species; *P < *0.001). Based on the COI-COII genetic information, 9 haplotypes (belonging to subspecies *A. cerana cerana*) of *A. cerana* and 6 haplotypes (belonging to 5 subspecies) of *A. mellifera* were identified in this study (Fig. 2f). Regardless of the substantial diversification of honeybee haplotypes across sampling sites (Fig. 2f), no significant influences of haplotype were observed on either honeybee gut bacterial diversity (two-way analysis of variance, *P > *0.1; Table S1a) or community composition (partial Mantel test, *P > *0.3; Table S1b) for both *A. cerana* and *A. mellifera*. This result did not totally dismiss the role of honeybee genetic diversification in influencing gut microbiota but suggested that the honeybee genetic variance at the subspecies or haplotype level is not enough to resist the stochastic or deterministic impact of other processes and factors, e.g., drift, priority effect, geographical location, and bacterial species pool (8, 25–27), to drive substantial change in gut bacterial communities.

10.1128/mBio.00751-21.1 FIG S1 Variance in the COI-COII region of honeybee mitochondrial DNA (mtDNA) can reflect the honeybee whole genomic mitochondrion genetic diversification. Download FIG S1, PDF file, 0.9 MB. Copyright © 2021 Ge et al. 2021 Ge et al. https://creativecommons.org/licenses/by/4.0/ This content is distributed under the terms of the Creative Commons Attribution 4.0 International license.

10.1128/mBio.00751-21.8 TABLE S1 Relative influence of honeybee haplotype and geography on the gut bacterial alpha diversities (a) and community composition (b). Download Table S1, DOCX file, 0.04 MB. Copyright © 2021 Ge et al. 2021 Ge et al. https://creativecommons.org/licenses/by/4.0/ This content is distributed under the terms of the Creative Commons Attribution 4.0 International license.

### Geography affects honeybee gut bacterial communities.

**FIG 3:**
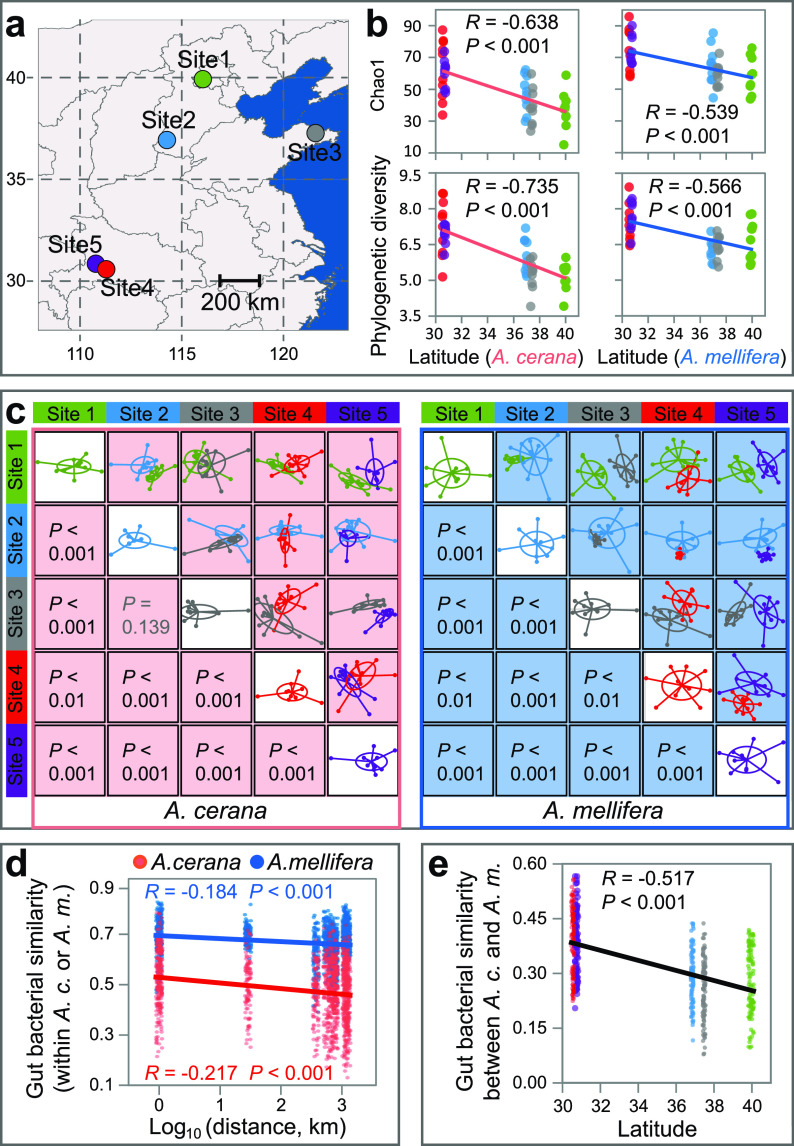
Effects of geography on *A. cerana* and *A. mellifera* gut bacterial communities. (a) The sampling sites. (b) The latitudinal patterns of honeybee gut bacterial diversity. (c) Nonmetric multidimensional scaling (NMDS) profiling illustrated geographical site effects on honeybee gut bacterial communities. *P* values were adjusted with the BH method. (d) The distance-decay relationship between geographical distance and community similarity of either *A. cerana* or *A. mellifera* gut microbiota. (e) The community similarity between *A. cerana* and *A. mellifera* gut bacterial communities negatively correlated with latitude.

Although not as important as host species as the major determinant, the influence of geography on honeybee gut microbiota cannot be ruled out (21, 22). Previous studies illustrated the significant influence of geography (or environment), which is secondary to host species (21, 28), but did not look into the relative role of geography and host genetic identity within the same host species. This study revealed that the role of geography overwhelms haplotype identity in affecting the gut bacterial diversity and community composition of both *A. cerana* and *A. mellifera* (Table S1a and b). The overwhelming role of environment over host genetic identity (within the same host species) was also observed in shaping human gut microbiota (29). In terms of alpha diversity, both *A. cerana* and *A. mellifera* presented similar patterns, with honeybees at low latitudes and, thus, high temperatures and precipitation levels (Fig. 3a and Table S2), and harbored gut bacterial communities with higher richness and phylogenetic diversity. This pattern was elucidated by the significantly negative correlations between diversity indices and latitude (*P < *0.001; Fig. 3b) and the significantly positive correlations between diversity indices and mean annual temperature (MAT) (*P < *0.001; Fig. S2a) and mean annual precipitation (MAP) (*P < *0.001; Fig. S2b). As a result of the latitude-diversity relationship, the diversity of local core gut bacteria, which take the main responsibility for the health and fitness of local hosts (2, 3, 5), also significantly correlated with latitude, MAT, and MAP (*P < *0.05; Fig. S3). There also were significantly positive correlations between *A. cerana* and *A. mellifera* gut bacterial diversity across geographical sites (*P < *0.001; Fig. S4).

10.1128/mBio.00751-21.2 FIG S2 Chao1 richness and phylogenetic diversity of *A. cerana* and *A. mellifera* gut bacterial communities positively correlated with mean annual temperature (MAT) and mean annual precipitation (MAP). Download FIG S2, PDF file, 1.1 MB. Copyright © 2021 Ge et al. 2021 Ge et al. https://creativecommons.org/licenses/by/4.0/ This content is distributed under the terms of the Creative Commons Attribution 4.0 International license.

10.1128/mBio.00751-21.3 FIG S3 Local core gut bacterial species (OTUs at 97% identity) number significantly correlates with latitude, mean annual temperature (MAT), and mean annual precipitation (MAP) for both *A. cerana* and *A. mellifera*. Download FIG S3, PDF file, 0.3 MB. Copyright © 2021 Ge et al. 2021 Ge et al. https://creativecommons.org/licenses/by/4.0/ This content is distributed under the terms of the Creative Commons Attribution 4.0 International license.

10.1128/mBio.00751-21.4 FIG S4 Positive correlations between *A. cerana* and *A. mellifera* gut bacterial diversity. Download FIG S4, PDF file, 1 MB. Copyright © 2021 Ge et al. 2021 Ge et al. https://creativecommons.org/licenses/by/4.0/ This content is distributed under the terms of the Creative Commons Attribution 4.0 International license.

10.1128/mBio.00751-21.9 TABLE S2 Site information. Download Table S2, DOCX file, 0.04 MB. Copyright © 2021 Ge et al. 2021 Ge et al. https://creativecommons.org/licenses/by/4.0/ This content is distributed under the terms of the Creative Commons Attribution 4.0 International license.

Since the latitudinal pattern of honeybee gut bacterial diversity was repeatedly observed for both *A. cerana* and *A. mellifera*, this pattern, i.e., honeybees at low latitudes possessing higher gut bacterial diversity than those at high latitudes, might be extrapolated to other honeybee species, although further studies are still needed to test this hypothesis. Likewise, in plants, animals, and free-living microbes, it is often reported that low latitudes harbor higher biodiversity than high latitudes, probably due to the higher productivity (productivity hypothesis), the more diverse niches (niche hypothesis), or the faster speciation (metabolism hypothesis) at low latitudes (30–32). However, few studies directly test whether animal-associated bacterial diversity also shows a latitudinal pattern. Our results provide the first evidence that honeybee gut bacterial diversity also presents a latitudinal pattern.

Besides diversity, honeybee gut bacterial composition also presented a latitudinal pattern. The NMDS profiling showed that, for both *A. cerana* and *A. mellifera*, the gut bacterial communities at different sites were significantly different from each other (*P < *0.001 except for *A. cerana* between sites 2 and 3; Fig. 3c). In particular, we found significant distance-decay relationships between geographical distances and gut bacterial similarities for both *A. cerana* and *A. mellifera* (*P < *0.001; Fig. 3d), evidencing the geographical patterns of honeybee gut bacterial communities. The distance-decay relationship was also found for mammalian gut microbiota (33).

Although the diversity and composition of both *A. cerana* and *A. mellifera* gut bacterial communities presented similar latitudinal patterns, *A. cerana* gut bacterial communities changed faster than *A. mellifera* along latitudinal gradient and geographical distance, as reflected by the steeper slopes of latitude-diversity relationships (*P < *0.05; Fig. 3b) and distance-decay relationships (*P < *0.01; Fig. 3d) with *A. cerana* than *A. mellifera*. The microbiota of the native *A. cerana* may have been influenced by the local environment for a far longer time than the newcomer *A. mellifera*, e.g., millions versus hundreds of years (5, 34). Additionally, the community similarities of gut bacteria between *A. cerana* and *A. mellifera* from the same geographical location negatively correlated with latitude (*R* = −0.52; *P < *0.001; Fig. 3e) and positively correlated with MAT (*R *= 0.54; *P < *0.001; Fig. S5a) and MAP (*R *= 0.50; *P < *0.001; Fig. S5b). These relationships were still significant after controlling the gut bacterial diversity (with latitude, *R* = −0.15; MAT, *R *= 0.18; MAP, *R *= 0.15; *P < *0.01; Fig. S6a to c), indicating that the observed latitudinal pattern of gut bacterial community similarity between *A. cerana* and *A. mellifera* was not solely derived from the latitudinal pattern of gut bacterial diversity (35). The higher gut bacterial community similarity between *A. cerana* and *A. mellifera* at low latitude may be the result of higher temperature, as high temperature tends to accelerate the community exchange and, thus, facilitates the role of the same local environmental factors in converging gut bacterial communities.

10.1128/mBio.00751-21.5 FIG S5 Community similarity between *A. cerana* and *A. mellifera* gut microbiota positively correlated with mean annual temperature (MAT) and mean annual precipitation (MAP). Download FIG S5, PDF file, 0.3 MB. Copyright © 2021 Ge et al. 2021 Ge et al. https://creativecommons.org/licenses/by/4.0/ This content is distributed under the terms of the Creative Commons Attribution 4.0 International license.

10.1128/mBio.00751-21.6 FIG S6 Partial correlations manifest that the gut bacterial similarity between *A. cerana* and *A. mellifera* (at same site) still significantly correlated with latitude, mean annual temperature (MAT), and mean annual precipitation (MAP) after controlling the gut bacterial alpha diversity. Download FIG S6, PDF file, 0.7 MB. Copyright © 2021 Ge et al. 2021 Ge et al. https://creativecommons.org/licenses/by/4.0/ This content is distributed under the terms of the Creative Commons Attribution 4.0 International license.

### Host species dominates over geographical site in shaping honeybee gut bacterial communities.

**FIG 4:**
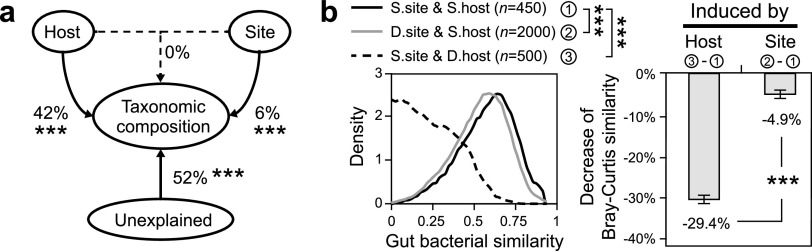
Relative contribution of host species and geographical site to honeybee gut bacterial communities. (a) Variance partitioning analysis (VPA) revealed that host species had higher effects on gut bacterial communities than geographical site. (b) The variations of host species and geographical site decreased the similarities of honeybee gut bacterial communities, while the decrease induced by host species was significantly larger than by geographical site. Error bars were 95% confidence intervals. ***, *P < *0.001.

Although both host species (Fig. 2) and geographical site (Fig. 3) altered honeybee gut bacterial communities, the result of variance partition analysis (VPA) revealed that host species had a stronger effect on gut bacterial communities than geographical site (42% versus 6%; Fig. 4a and Fig. S7). The dominant role of host species in governing honeybee gut bacterial communities was also supported by directly comparing community similarities: comparing the community similarities within the same host and site, the community similarities between host species and between geographical sites were significantly decreased (*P < *0.001; Fig. 4b), and the decrease induced by host species was significantly greater than that by geographical site (|−29.4%| > |−4.9%|; *P < *0.001; Fig. 4b). Previous studies have examined the effects of various factors, e.g., host attribute, sampling site, diet, and contaminant, on host-associated bacterial communities (8, 29, 36–39), but little is known about their relative quantitative importance. Here, we demonstrate that host species dominates over geographical site in shaping honeybee gut bacterial communities and, thus, provides insights into the quantitatively relative contribution of different factors in governing honeybee gut microbiota.

10.1128/mBio.00751-21.7 FIG S7 Pairwise comparison illustrates that host species dominates over geographical site in governing honeybee gut bacterial communities by the analyses of nonmetric multidimensional scaling (NMDS), permutational multivariate analysis of variance (PERMANOVA), and variance partition analysis (VPA). Download FIG S7, PDF file, 0.4 MB. Copyright © 2021 Ge et al. 2021 Ge et al. https://creativecommons.org/licenses/by/4.0/ This content is distributed under the terms of the Creative Commons Attribution 4.0 International license.

### Ecological processes governing honeybee gut bacterial communities.

**FIG 5:**
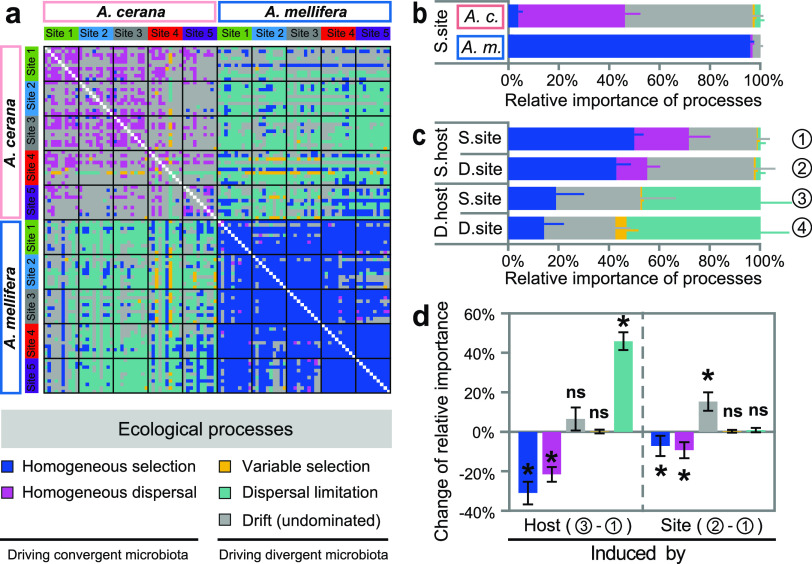
Ecological processes driving the assembly and shift of honeybee gut bacterial communities. (a) Ecological processes between paired samples. (b) The relative importance of ecological processes in governing the gut bacterial communities of *A. cerana* and *A. mellifera* without considering the effects of host species or geographical site. (c) The relative importance of ecological processes in governing honeybee gut bacterial communities of the same host at the same site (S.host and S.site), the same host at different sites (S.host and D.site), different hosts at the same site (D.host and S.site), and different hosts at different sites (D.host and S.site). (d) Host- and site-induced change of ecological processes’ relative importance in governing the shift of honeybee gut bacterial communities. Error bars were 95% confidence intervals. *, *P < *0.05; ns (not significant), *P > *0.05.

In an effort to gain insight into the mechanisms underlying the host-associated and geographical patterns of honeybee gut bacterial communities, we evaluated the phylogenetic signals of five ecological processes that can cause communities to converge (homogeneous selection and homogeneous dispersal), diverge (variable selection and dispersal limitation), and experience stochastic variation (drift [40], also referred to as the undominated process [41]; Fig. 1 and 5a). The phylogenetic signals were evaluated by calculating the deviation between empirically observed β-mean nearest taxon distance (βMNTD) or Bray-Curtis distance and the null distribution; the βMNTD deviation and Bray-Curtis deviation were denoted as β-nearest taxon index (βNTI) and Raup-Crick index (RC), respectively (19, 40–42). βNTI of <−2 or >2 indicates significantly less or greater community turnover (than null expectations) that was caused by the selection of similar (homogeneous selection) or contrast (variable selection) environmental conditions, while −2 ≤ βNTI ≤ 2 indicates that selection does not affect community turnover (40). For those −2 ≤ βNTI ≤ 2, RC of <−0.95 or >0.95 indicates significantly less or greater community turnover (than null expectations) that was caused by high dispersal (homogeneous dispersal) or restricted dispersal (dispersal limitation), while −0.95 ≤ RC ≤ 0.95 indicates stochastic drift (40) or the undominated process (neither dispersal nor selection as the primary determinant) (41).

First, we examined the processes that govern the composition of honeybee gut bacterial communities without considering the effects of host species and geographical site. The results showed that the processes were different for *A. cerana* and *A. mellifera*: homogeneous dispersal (relative contribution, 42.2%) was the major process driving *A. cerana* gut bacterial communities to convergence, while homogeneous selection (96.0%) was the major process driving *A. mellifera* gut bacterial communities to convergence (Fig. 5b). Compared to *A. cerana*, *A. mellifera* is characterized by larger body/colony size and gut capacity, wider foraging range, and a distinct immune system (21), which might be the latent reasons underlying the different ecological processes between the two species. Although drift (or undominated process) was the major process driving the interindividual variation of gut bacterial communities for both *A. cerana* and *A. mellifera*, its relative contribution was much higher for *A. cerana* (50.7%) than *A. mellifera* (3.1%; Fig. 5b). These results could explain the relatively higher fluctuation of *A. cerana* gut bacterial communities than those of *A. mellifera* (Fig. 2c). Here, because the host species and geographical site were not included in the analyses, the homogeneous dispersal likely reflected the gut bacterial transmission between honeybee individuals within the same honeybee species or from the same environmental microbial pool (25, 43), which mediated the similarity and stability of *A. cerana* gut bacterial communities across individuals, while the homogeneous selection probably reflected the selection of the same inner gut environment or external environment (26), which maintained the similarity and stability of *A. mellifera* gut bacterial communities.

### Host species and geographical site differentiate honeybee gut bacterial communities by changing the relative contribution of different processes.

When we introduced host as an additional factor, we found that host species significantly altered the relative importance of different ecological processes in shaping honeybee gut bacterial communities. For example, the processes causing the gut bacterial communities to converge, i.e., homogeneous selection and homogeneous dispersal, were significantly decreased (*P < *0.05), while the process causing the gut bacterial communities to diverge, i.e., dispersal limitation, was significantly increased (*P < *0.05; Fig. 5c and d). This result suggested that, on the one hand, within the same honeybee species, the homogeneous dispersal caused by the frequent interaction between social members (44) or the homogenous selection by the conservative intestinal niches (45) converged on the gut bacterial communities (Fig. 1a). On the other hand, between different honeybee host species, the interhost isolation and the interhost neutrality-based dispersal limitation, modulated by the host immune systems and microbe-filtering organs (46, 47), differentiated the gut bacterial communities (Fig. 1b). Consistent with this, a previous study showed that the microbial communities from a wide range of host organisms, particularly invertebrates, are under the control of neutrality (48). Because *A. cerana* and *A. mellifera* were geographically isolated from each other for millions of years before *A. mellifera* was introduced into the native region of *A. cerana* in the early 1900s (5, 34), the dispersal limitation may also reflect the heritable effects of historically geographical isolation between *A. cerana* and *A. mellifera.* Therefore, the neutrality-based dispersal limitation derived from interhost or historically geographical isolation (Fig. 5c and d), in association with long-term coevolution between eukaryotic hosts and prokaryotic symbionts, diverged in the gut microbiota between *A. cerana* and *A. mellifera* (Fig. 2).

The geography also affected the relative importance of different ecological processes in shaping honeybee gut bacterial communities. Similar to the effects of host species, when geographical site was involved in the analyses, the processes converging the gut bacterial communities, i.e., homogeneous selection and homogeneous dispersal, were significantly decreased (*P < *0.05), while the process diverging the gut bacterial communities was significantly increased (*P < *0.05; Fig. 5c and d). However, unlike the effects of host species, stochastic drift (or undominated process) rather than dispersal limitation was the major enhanced process that caused community divergence (Fig. 5d). These results suggested that geographical site mainly functioned on enhancing stochastic drift and suppressing homogeneous selection and homogeneous dispersal, by which divergent bacterial communities across geographical sites were shaped (Fig. 3).

Models derived from neutral theory have demonstrated that drift is sufficient to differentiate communities over space, since chance birth and death events differ among geographical locations (15). The development direction of gut bacterial communities driven by stochastic drift was uncertain, but within a specific geographical site, the homogeneous interactions among individual honeybees drove gut bacterial communities toward a single direction and, thus, shaped relatively convergent gut microbiota. However, the free interactions between honeybees located at different geographical sites were restricted; thus, stochastic drift drove gut bacterial communities toward divergent compositions whose degree was dependent on geographical distance and the associated evolutionary history. Therefore, honeybee gut bacterial communities would be more similar between nearby sites than between distant sites, as elucidated by the distance-decay relationships (Fig. 3d).

Although variations in host species and geographical site enhanced the processes that diverge gut bacterial communities and inhibited the processes that converge gut bacterial communities, the host-induced change was three times higher than the site-induced change (Fig. 5d), which can explain the higher effects of host species than geography on honeybee gut bacterial communities (Fig. 4 and Fig. S7).

### Historical stochasticity drives the coevolution of honeybee gut microbiota.

**FIG 6:**
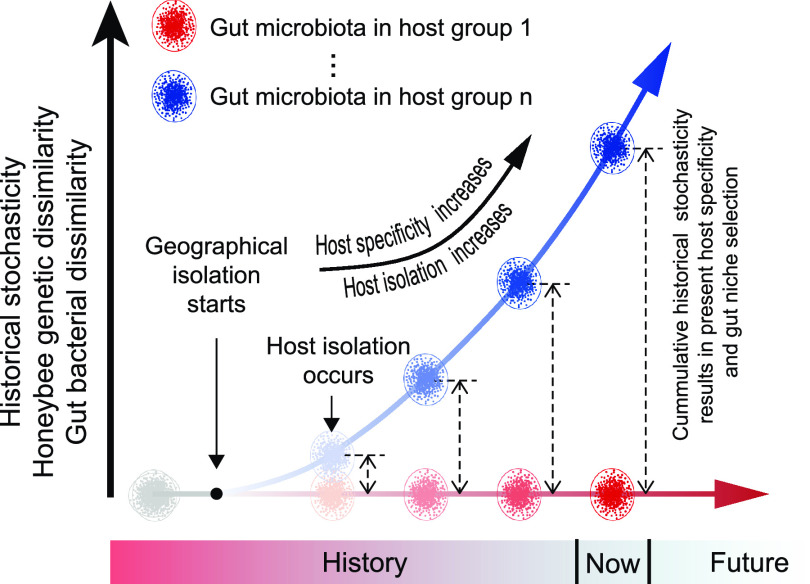
Historical stochasticity engines the coevolution of honeybee gut microbiota.

Although geography has been dismissed as the major factor influencing honeybee gut microbiota (21, 22), this study established the essential role of geography in pushing the coevolution of honeybee and gut microbiota. Specifically, at the beginning of the coevolution, where host genetic isolation does not exist, the geographical isolation enables the stochastic processes to drive host genetic diversification as well as random changes of gut microbiota (25, 43, 49), processes of which were further regulated by the geographical factors (e.g., local environment, latitude, MAT, and MAP) (30, 31). After a long period of geographical isolation, when the cumulative historical stochasticity causes substantial change of host genetic identity, the host genetic isolation occurs and results in different host-bacterium specificity and niche identity (Fig. 6). Restricted by host specificity and niche conservatism, only specific microbes that benefit their eukaryotic host can be deterministically gathered to assemble the optimal gut microbiota (45, 50), resulting in honeybee gut bacterial community conservation along each coevolution path (Fig. 6). Therefore, across the whole coevolution history of honeybee gut bacterial communities, the neutrality-based stochastic processes tend to be the main forces driving coevolution, and the deterministic processes determine the direction of coevolution.

### Conclusions.

Host species dominates over geography in shaping honeybee gut bacterial communities. Although secondary to the role of the host, the role of geography in shaping the honeybee gut microbiota cannot be neglected. The host-based and geographical patterns of honeybee gut bacterial communities tend to be shaped by the coworking of different ecological forces. This study elucidates that neutrality (dispersal limitation and stochastic drift) rather than selection (variable selection) underlying honeybee gut bacterial community composition is enhanced by variations in host species and geography and provides insights into the dominant role of neutrality over selection in differentiating honeybee gut microbiota across evolutionary time.

## MATERIALS AND METHODS

### Experimental design.

*A. cerana* and *A. mellifera*, the two most widely studied model species of honeybee (9, 21), were collected from five geographical sites (distance of up to 1,000 km) across northern and southern China in June and July 2017. The five geographical sites were selected because they represented a latitudinal gradient with distinct temperature, rainfall, and environmental conditions (see Table S2 in the supplemental material). The healthy adult worker honeybees, 20 to 30 days after breaking comb cells and with similar body sizes, of *A. cerana* and *A. mellifera* were simultaneously collected from four hives at each site. Managed colonies were chosen for this study, because the age, health state, hive material, and forage conditions were under control for both *A. cerana* and *A. mellifera* at each sampling site. With each honeybee species, 10 individuals were pooled from samples collected from each site to achieve 10 experimental replicates per host species and geographical site. The guts of the worker honeybees were carefully sampled with sterile forceps into 1.5-ml centrifuge tubes and stored at −80°C.

### DNA extraction.

A FastDNA spin kit (MP Biomedicals, CA, USA) was used to extract the genomic DNA from honeybee gut samples. The extracted DNA was dissolved in 50 μl Tris-EDTA (TE) buffer, quantified by a NanoDrop 2000 UV-visible (UV-vis) spectrophotometer (NanoDrop, DE, USA), and qualitatively evaluated by gel electrophoresis.

### Analysis of honeybee gut bacterial diversity.

With each gut DNA sample, PCR for the V3-V4 region of bacterial 16S rRNA gene was conducted in triplicate with the primer set 338F (5′-ACTCCTACGGGAGGCAGCA-3′) and 806R (5′-GGACTACHVGGGTWTCTAAT-3′). The oligonucleotides of 6-bp barcodes were fused to the forward and reverse primers to assign sequences to samples postsequencing. PCR was carried out in 50-μl reaction mixtures containing 2 U *Taq* DNA polymerase (TaKaRa, Tokyo, Japan), 5 μl deoxynucleotide triphosphates (dNTPs) (2.5 mM), 2 μl each primer (5 μM), and 10 ng template DNA. The PCR procedures were 95°C for 3 min, 25 cycles of 95°C for 30 s, 55°C for 30 s, and 72°C for 45 s, with a final extension for 10 min at 72°C. A negative-control experiment and agarose gel electrophoresis were conducted to ensure no contamination, nonspecific amplification, or dimers. The triplicate PCR products of each gut DNA sample were pooled and purified using the QIAquick PCR purification kit (Qiagen, CA, USA) and quantified using a NanoDrop 2000 UV-vis spectrophotometer (NanoDrop, DE, USA). The purified PCR products from all samples were normalized in equimolar amounts before sequencing.

High-throughput sequencing was performed with the Illumina MiSeq PE300 sequencing platform (Illumina Inc., CA, USA). The sequences were processed using the Quantitative Insights Into Microbial Ecology (QIIME) pipeline (51). In brief, adapter and barcodes were truncated, and sequences of low quality (with quality score below 20, length fewer than 350 bp, or ambiguous bases) were screened. Rare sequences with fewer than 2 reads were removed before OTU clustering to eliminate potentially artificial taxa. The remaining high-quality sequences were clustered into OTUs using UPARSE (52) at the 97% identity threshold. The representative sequences were aligned by PyNAST (51), and the phylogenetic tree was constructed using FastTree (http://www.microbesonline.org/fasttree/). The OTUs were assigned to taxonomy against the Silva 132 database (http://www.arb-silva.de/download/archive/qiime/). A total of 2,680,274 sequences were generated, and the sequencing depth of the 100 gut samples ranged from 19,602 to 32,465, with a median of 26,597. Because uneven sequencing depth across samples may confuse the results of community comparisons, we rarified the original OTU table 100 times to increase the reliability of community comparisons across samples (19,600 sequences per sample). The rarefied OTU tables were used for the OTU-based community analysis. The local core OTUs were defined as the OTUs with relative abundance of  ≥0.1% and occurrence of ≥80% (e.g., observed in at least 8 of the 10 samples collected from a specific geographical location), and an OTU was further defined as a regional core OTU if it was identified as a local core OTU at all sampling sites.

### Identification of honeybee haplotype diversity.

The comparative analysis of the mitochondrial DNA (mtDNA) variance in the COI-COII region is extensively used to identify the honeybee subspecies and genetic diversity (53). For each gut DNA sample, the COI-COII region of mtDNA of honeybee was amplified with the primer pair prC1C2-L (5′-CCACGACGTTATTCAGACTATCCA-3′) and prC1C2-R (5′-CAT ATGATCAATATCATTGATGACCAA-3′) (53). PCR was carried out in 50-μl reaction mixtures containing 2 U *Taq* DNA polymerase (TaKaRa, Tokyo, Japan), 5 μl dNTPs (2.5 mM), 1 μl each primer (5 μM), and 10 ng template DNA. The PCR procedures were 94°C for 3 min, 33 cycles of 94°C for 30 s, 56°C for 45 s, and 72°C for 1 min, and a final extension for 10 min at 72°C. PCR products were purified using the agarose gel DNA purification kit (Qiagen, CA, USA) and sequenced using an ABI 3730xl sequencer (Applied Biosystems, CA, USA). The nucleotide sequences were processed using Chromas (http://technelysium.com.au/wp/chromas/) and DNASTAR (https://www.dnastar.com/). Sequences were aligned using the Clustal W method (54), and the maximum likelihood tree, based on the Tamura-Nei model (55), was constructed using MEGA (https://megasoftware.net/). Honeybee subspecies were identified by comparing the haplotype sequences to the GenBank references using BLAST (http://blast.ncbi.nlm.nih.gov/Blast.cgi).

### Estimation of ecological processes.

The estimation of ecological processes was performed according to Stegen et al. (40). First, both βNTI and RC were calculated using null models (1,000 randomizations), with the consideration of OTU abundances (log transformed) (35). We then incorporated βNTI and RC to estimate the relative strength of homogeneous selection (βNTI < −2), variable selection (βNTI > 2), homogeneous dispersal (RC < −0.95 and |βNTI| ≤ 2), dispersal limitation (RC > 0.95 and |βNTI| ≤2), and drift (also refers to as the undominated process; |RC| ≤0.95 and |βNTI| ≤2) in governing the composition of the gut bacterial community (40–42). We conducted this procedure based on the 100 rarefied OTU tables.

### Statistical analysis.

NMDS based on Bray-Curtis distance was conducted with the VEGAN package (56) in R to visualize the community differences among samples. PERMANOVA was performed using adonis in the VEGAN package (56) to test whether the effects of host species and the geographical site on honeybee gut bacterial communities were significant. VPA was conducted with the VEGAN package (56) to examine the relative contribution of honeybee species, geographical site, and honeybee haplotype in differentiating gut bacterial communities, and the significance of partitioned fractions was tested by performing a permutation test for distance-based redundancy analysis using anova.cca in the VEGAN package (56). The significance of the correlation or partial correlation between distance matrices was tested using mantel or partial.mantel in the VEGAN package (56). Student's *t* test was used to test the difference of variables, e.g., bacterial diversity indices between host species and Bray-Curtis similarities between different groups. Bootstrapping (1,000 times) followed by *t* test was conducted to test the differences of regressed slopes from zero (57) and the pairwise differences (58); the same method was also conducted to test whether the host and site induced changes, e.g., the relative importance of processes and community similarity were significantly different from zero, and calculated the 95% confidence intervals (57). *P* values for multiple testing were adjusted with the BH (Benjamini and Hochberg) method (59) using the p.adjust function in R.

### Data availability.

Sequences of bacterial 16S rRNA genes have been deposited in the National Center for Biotechnology Information (NCBI) Sequence Read Archive (SRA) with the accession number PRJNA505716. The COI-COII haplotype sequence data are provided in Dataset S1 in the supplemental material.

10.1128/mBio.00751-21.10 DATA SET S1 The COI-COII haplotype sequences of the 100 honeybee samples. Download Data Set S1, XLSX file, 0.02 MB. Copyright © 2021 Ge et al. 2021 Ge et al. https://creativecommons.org/licenses/by/4.0/ This content is distributed under the terms of the Creative Commons Attribution 4.0 International license.
