# Supplementary material for: Host Species and Geography Differentiate Honeybee Gut Bacterial Communities by Changing the Relative Contribution of Community Assembly Processes
Source: mBio. 2021 Jun 1;12(3):e00751-21. doi: 10.1128/mBio.00751-21 (PMC8262996; doi:10.1128/mBio.00751-21)
Supplement: FIG S1 [file mbio.00751-21-sf001.pdf]

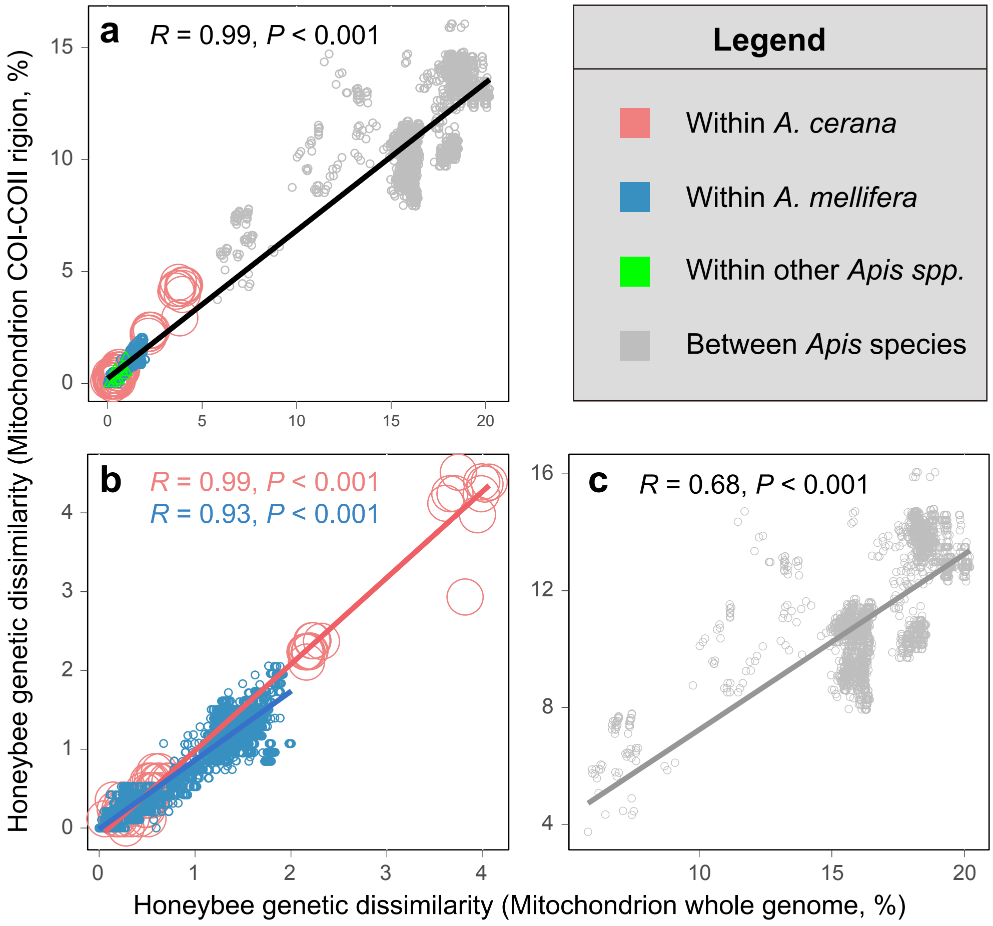

Fig. S1 Variance in the COI-COII region of honeybee mitochondrial DNA (mtDNA) can reflect the honeybee whole genomic mitochondrion genetic diversification. X-axis, the pairwise honeybee genetic dissimilarities (red: between haplotypes of *A. cerana*； blue: between haplotypes of *A. mellifera*; green: haplotypes of other *Apis* species; gray: between *A. cerana and A. mellifera*) based on honeybee whole genomic mitochondrion sequences. Y-axis, the pairwise honeybee genetic dissimilarities based on the COI-COII region subset cut from the same whole genomic mitochondrion sequences. a, Linear regression based on all pairs of genetic dissimilarities. b, Linear regressions based on pairwise genetic dissimilarities among haplotypes of *A. cerana* (red) or *A. mellifera* (blue:). c, Linear regression based on pairwise genetic dissimilarities between different honeybee species. *R*: Pearson's correlation coefficient; *P*: significance of correlation assessed by mantel test or mantel-like permutation test (1) by shuffling the columns and rows of dissimilarity matrices. [Processes for calculating the genetic dissimilarities in X-axis and Y-axis: All available whole genomic mitochondrion sequences of *Apis* were downloaded from NCBI database, followed by length filtering (> 10000 bp) and dereplication. A total of 113 unique mitochondrial whole genome sequences were retained (*A. cerana*, 11; *A. mellifera*, 86; *A. florea*, 3; *A. laboriosa*, 2; *A. dorsata*, 3; *A. nigrocincta*, 3; *A. andreniformis*, 3; *A. koschevnikovi*, 2), and then aligned with MEGA (https://megasoftware.net/) using the Clustal W method (2); The COI-COII region, between the primer pair prC1C2-L (5'-CCA CGA CGT TAT TCA GAC TAT CCA-3') and prC1C2-R (5'-CAT ATG ATC AAT ATC ATT GAT GAC CAA-3') (3), in each retained mitochondrial whole genome were

cut out, and aligned. Finally the honeybee genetic dissimilarities based on the whole genome and the COI-COII region of mitochondrion were calculated respectively using MEGA (https://megasoftware.net/).]

1. Legendre P, Borcard D, Peres-Neto PR. 2005. Analyzing beta diversity: Partitioning the spatial variation of community composition data. *Ecol Monogr* **75**:435-450. https://doi.org/10.1890/05-0549

2. Thompson JD, Higgins DG, Gibson TJ. 1994. CLUSTAL W: Improving the sensitivity of progressive multiple sequence alignment through sequence weighting, position-specific gap penalties and weight matrix choice. *Nucleic Acids Res* **22**:4673-4680. https://doi.org/10.1093/nar/22.22.4673

3. Cherevatov OV, Panchuk II, Kerek SS, Volkov RA. 2019. Molecular diversity of the COI–COII spacer region in the mitochondrial genome and the origin of the Carpathian bee. *Cytol Genet* **53**:276-281. https://doi.org/10.3103/S0095452719040030
