# Supplementary material for: Host Species and Geography Differentiate Honeybee Gut Bacterial Communities by Changing the Relative Contribution of Community Assembly Processes
Source: mBio. 2021 Jun 1;12(3):e00751-21. doi: 10.1128/mBio.00751-21 (PMC8262996; doi:10.1128/mBio.00751-21)
Supplement: TABLE S1 [file mbio.00751-21-st001.docx]

**Table S1 Relative influence of honeybee haplotype and geography on the gut bacterial alpha diversities (a) and community composition (b)**

| **a** Two-way ANOVA (analysis of variance) showed that honeybee haplotype has no influence on honeybee gut bacterial alpha diversities | | | | | | | | | | |  |
| --- | --- | --- | --- | --- | --- | --- | --- | --- | --- | --- | --- |
| Host species | Bacterial diversity | | Tested factor | | Mean square | | | F value | *P* value | |  |
| *A. cerana* | Chao1 | | **Geographical site** | | **529.07** | | | **3.52** | **.039** | |  |
|  |  |  | Honeybee haplotype | | 247.47 | | | 1.65 | .161 | |  |
|  | Phylogenetic diversity | | **Geographical site** | | **2.61** | | | **4.79** | **.014** | |  |
|  |  |  | Honeybee haplotype | | .627 | | | 1.15 | .353 | |  |
| *A. mellifera* | Chao1 | | Geographical site | | 165.18 | | | 1.28 | .302 | |  |
|  |  |  | Honeybee haplotype | | 18.56 | | | .14 | .966 | |  |
|  | Phylogenetic diversity | | **Geographical site** | | **1.51** | | | **3.19** | **.034** | |  |
|  |  |  | Honeybee haplotype | | .03 | | | .06 | .992 | |  |
| **b** Partial Mantel test and variance partition analysis (VPA) showed more significant and higher relative impact of geography than honeybee haplotype identity on gut bacterial community | | | | | | | | | | | |
| Host species | | Tested factor | | Partial Mantel Test | | | Variance Partition Analysis | | | | |
|  |  |  |  | *R* | | *P* value | Relative impact | | | *P* value | |
| *A. cerana* | | **Geographical site** | | **0.162** | | **0.001** | **3.2%** | | | **0.031** | |
|  |  | Honeybee haplotype | | -0.014 | | 0.575 | 0.0% | | | 0.358 | |
|  |  | **Geographical distance** | | **0.207** | | **0.001** | **7.0%** | | | **0.001** | |
|  |  | Honeybee genetic distance | | -0.028 | | 0.603 | 0.0% | | | 0.327 | |
| *A. mellifera* | | **Geographical site** | | **0.220** | | **0.001** | **10.6%** | | | **0.001** | |
|  |  | Honeybee haplotype | | -0.097 | | 0.941 | 0.0% | | | 0.342 | |
|  |  | **Geographical distance** | | **0.189** | | **0.001** | **9.2%** | | | **0.001** | |
|  |  | Honeybee genetic distance | | -0.06 | | 0.679 | 0.0% | | | 0.512 | |

Notes: The *P* values (significance) of the variance partition results were tested by performing an ANOVA (analysis of variance) like permutation test for distance-based redundancy analysis using the function ‘anova.cca’ in the VEGAN package (1).

1. Oksanen J, Blanchet FG, Kindt R, Legendre P, Minchin RB, O'Hara PR, Simpson GL, Solymos P, Stevens MHH, Helene W. 2018. vegan: Community Ecology Package. R package version 2.5-2. <https://CRAN.R-project.org/package=vegan>
