# Supplementary material for: Host Species and Geography Differentiate Honeybee Gut Bacterial Communities by Changing the Relative Contribution of Community Assembly Processes
Source: mBio. 2021 Jun 1;12(3):e00751-21. doi: 10.1128/mBio.00751-21 (PMC8262996; doi:10.1128/mBio.00751-21)
Supplement: FIG S2 [file mbio.00751-21-sf002.pdf]

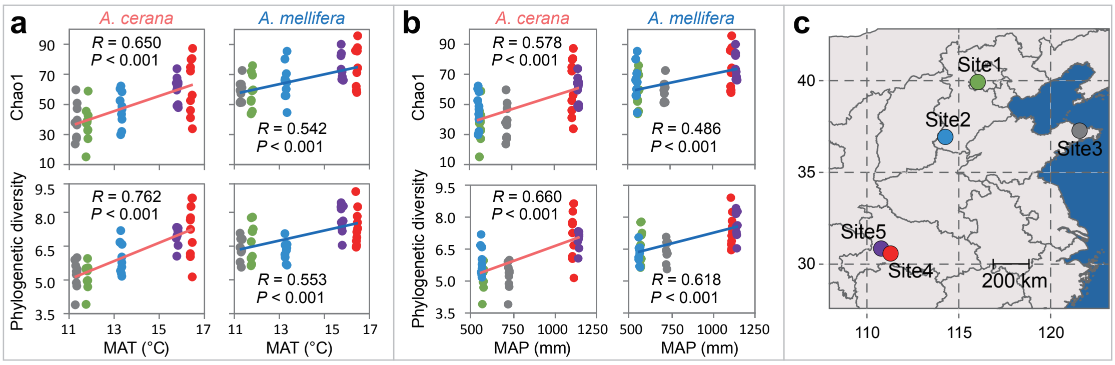

Fig. S2 Chao1 richness and phylogenetic diversity of *A. cerana* and *A. mellifera* gut bacterial communities positively correlated with mean annual temperature (MAT) and mean annual precipitation (MAP). **a**, Positive correlations between diversity indices and MAT. **b**, Positive correlations between diversity indices and MAP. **c**, The sampling sites.
