# Supplementary material for: Host Species and Geography Differentiate Honeybee Gut Bacterial Communities by Changing the Relative Contribution of Community Assembly Processes
Source: mBio. 2021 Jun 1;12(3):e00751-21. doi: 10.1128/mBio.00751-21 (PMC8262996; doi:10.1128/mBio.00751-21)
Supplement: FIG S3 [file mbio.00751-21-sf003.pdf]

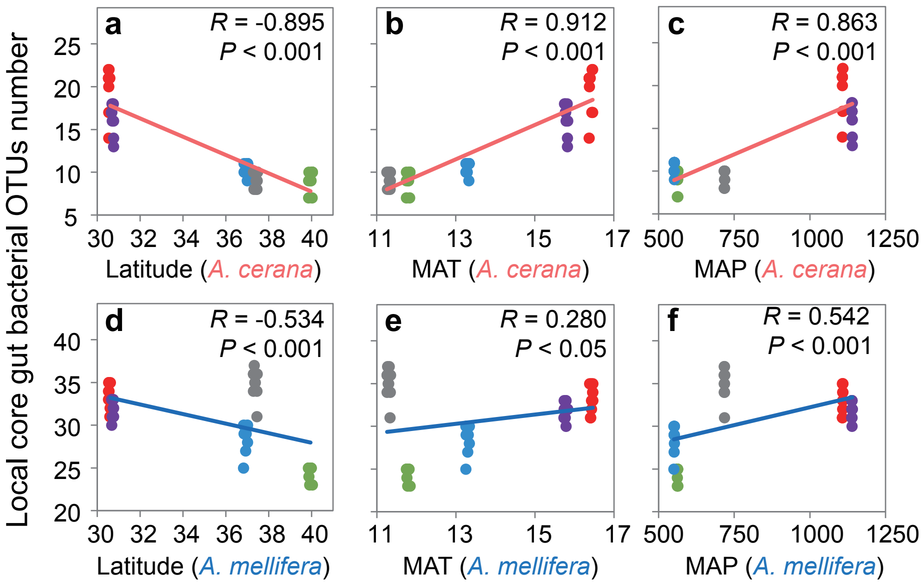

Fig. S3 Local core gut bacterial species (OTUs at 97% identity) number significantly correlates with latitude, mean annual temperature (MAT), and mean annual precipitation (MAP) for both *A. cerana* and *A. mellifera*. a-c, *A. cerana*. d-f, *A. mellifera*.
