# Supplementary material for: Host Species and Geography Differentiate Honeybee Gut Bacterial Communities by Changing the Relative Contribution of Community Assembly Processes
Source: mBio. 2021 Jun 1;12(3):e00751-21. doi: 10.1128/mBio.00751-21 (PMC8262996; doi:10.1128/mBio.00751-21)
Supplement: FIG S4 [file mbio.00751-21-sf004.pdf]

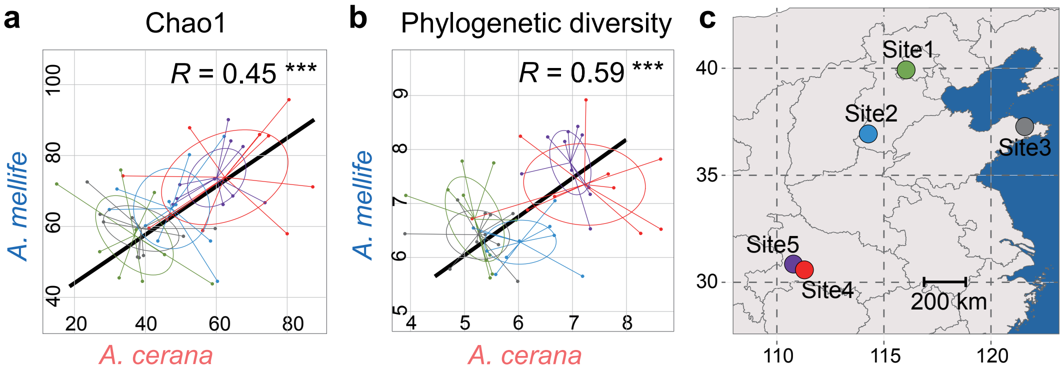

Fig. S4 Positive correlations between *A. cerana* and *A. mellifera* gut bacterial diversity. a, Chao1 richness. **b**, Phylogenetic diversity. **c**, The sampling sites. ***: $P < 0.001$. [Notes: Because 10 individuals were randomly sampled from each sampling site for both honeybee species (n_{sample} = 10 individuals * 5 sites * 2 hosts), within the same sampling site the 10 samples of *A. cerana* were not in strictly one-to-one correspondence with those of *A. mellifera*. To better estimate the Pearson correlation (n = 50) between *A. cerana* and *A. mellifera* gut bacterial diversity, the order of the gut bacterial diversity indices of 10 bees (of both hosts) within the same site were permuted for 1000 times to generate 1000 permuted diversity vectors, based on which 1000 Pearson correlation coefficients and *P* values were calculated. Finally, the mean Pearson correlation coefficient and the upper confidence interval (95%) of the *P* values were used.]
