# Supplementary material for: Host Species and Geography Differentiate Honeybee Gut Bacterial Communities by Changing the Relative Contribution of Community Assembly Processes
Source: mBio. 2021 Jun 1;12(3):e00751-21. doi: 10.1128/mBio.00751-21 (PMC8262996; doi:10.1128/mBio.00751-21)
Supplement: TABLE S2 [file mbio.00751-21-st002.docx]

**Table S2 Site information**

|  | Site 1 | Site 2 | Site 3 | Site 4 | Site 5 |
| --- | --- | --- | --- | --- | --- |
| Latitude | 39.94° N | 36.92° N | 37.38° N | 30.62° N | 30.67° N |
| Longitude | 115.96° E | 114.17° E | 121.4° E | 111.33° E | 111.04° E |
| Elevation (m) | 1670 | 1130 | 232 | 220 | 1450 |
| MAT (℃) | 11.79 | 13.29 | 11.30 | 16.42 | 15.78 |
| MAP (mm) | 562 | 552 | 717 | 1108 | 1139 |

Notes: The information of latitude, longitude, and elevation was collected with global position system (GPS) when sampling at each geographical site. The mean annual temperature (MAT) and mean annual precipitation (MAP) of each geographical site were calculated by Kriging interpolation based on the 5-year (2013-2017) mean annual temperature and precipitation grid dataset (0.5° * 0.5°) downloaded from National Meteorological Information Center (<http://data.cma.cn/>).
