# Supplementary material for: Host Species and Geography Differentiate Honeybee Gut Bacterial Communities by Changing the Relative Contribution of Community Assembly Processes
Source: mBio. 2021 Jun 1;12(3):e00751-21. doi: 10.1128/mBio.00751-21 (PMC8262996; doi:10.1128/mBio.00751-21)
Supplement: FIG S5 [file mbio.00751-21-sf005.pdf]

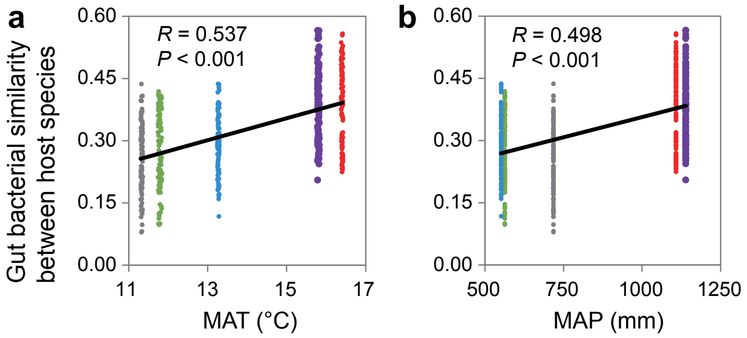

Fig. S5 Community similarity between *A. cerana* and *A. mellifera* gut microbiota positively correlated with mean annual temperature (MAT) and mean annual precipitation (MAP). a, Positive correlation with MAT. **b**, Positive correlation with MAP.
