# Supplementary material for: Host Species and Geography Differentiate Honeybee Gut Bacterial Communities by Changing the Relative Contribution of Community Assembly Processes
Source: mBio. 2021 Jun 1;12(3):e00751-21. doi: 10.1128/mBio.00751-21 (PMC8262996; doi:10.1128/mBio.00751-21)
Supplement: FIG S6 [file mbio.00751-21-sf006.pdf]

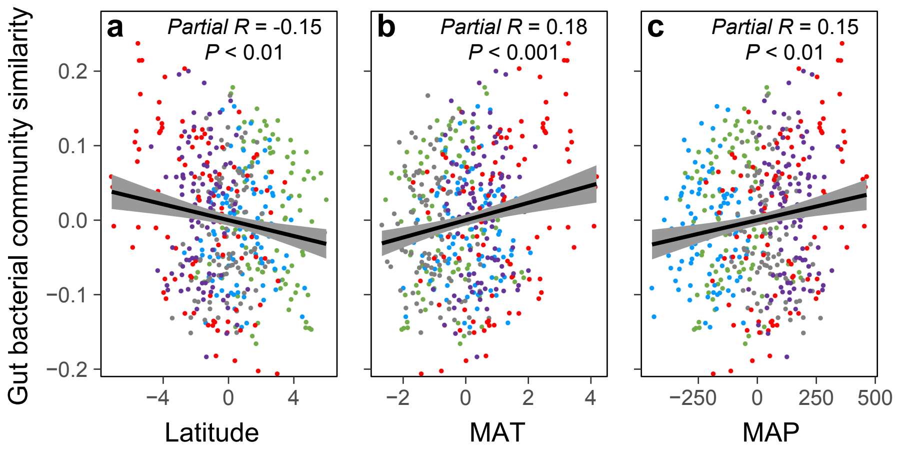

Fig. S6 Partial correlations manifest that the gut bacterial similarity between *A. cerana* and *A. mellifera* (at same site) still significantly correlated with latitude, mean annual temperature (MAT), and mean annual precipitation (MAP) after controlling the gut bacterial alpha diversity. X-axis, the residuals of the gut bacterial similarities after controlling the effect of bacterial alpha diversity (Means of the z-score of Chao1 and phylogenetic diversity); Y-axis, the residuals of geographical factors (in panel a: latitude, b: MAT, and c: MAP) after controlling the effect of bacterial alpha diversity. The significances of correlations were calculated by conducting mantel-like permutation test (n = 1000) by shuffling the columns and rows of matrices (1). [Notes: As the decreased or increased gut bacterial diversity with latitude, MAT and MAP (**Fig. 3b and Fig. S2a-b**) may also drive the significant latitudinal pattern of bacterial community similarity (2) between *A. cerana* and *A. mellifera* from same location (**Fig. 3e and Fig. S5a-b**). Thus we conducted partial correlations to test whether the correlations between the gut community similarity and geographical factors still exist after controlling the gut bacterial alpha diversity. The result showed that the gut community similarities were still significantly correlated with all the three geographical factors after controlling the gut bacterial diversities, which further consolidated the observed latitudinal pattern of the gut bacterial similarity between different honeybee species from the same geographical location.]

1. Legendre P, Borcard D, Peres-Neto PR. 2005. Analyzing beta diversity: Partitioning the spatial variation of community composition data. *Ecol Monogr* **75**:435-450. https://doi.org/10.1890/05-0549

2. Chase JM, Kraft NJB, Smith KG, Vellend M, Inouye BD. 2011. Using null models to disentangle variation in community dissimilarity from variation in α-diversity. *Ecosphere* **2**:art24. http://doi.org/10.1890/es10-00117.1
