# Supplementary material for: Host Species and Geography Differentiate Honeybee Gut Bacterial Communities by Changing the Relative Contribution of Community Assembly Processes
Source: mBio. 2021 Jun 1;12(3):e00751-21. doi: 10.1128/mBio.00751-21 (PMC8262996; doi:10.1128/mBio.00751-21)
Supplement: FIG S7 [file mbio.00751-21-sf007.pdf]

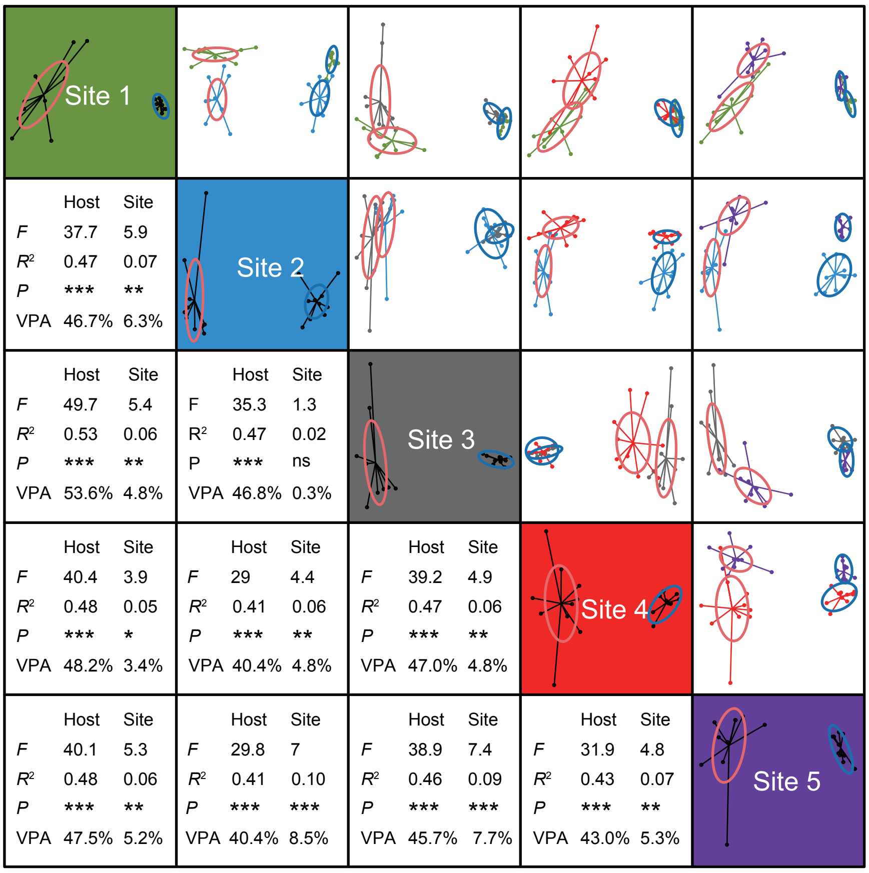

Fig. S7 Pairwise comparison illustrates that host species dominates over geographical site in governing honeybee gut bacterial communities by the analyses of non-metric multidimensional scaling (NMDS), permutational multivariate analysis of variance (PERMANOVA), and variance partition analysis (VPA). ***: P < 0.001; **: P < 0.01; *: P < 0.05; ns: P > 0.05. P values were adjusted with BH (Benjamini & Hochberg) method (1).

1. Benjamini Y, Hochberg Y. 1995. Controlling the false discovery rate: a practical and powerful approach to multiple testing. *J R Stat. Soc Ser B-Stat. Methodol* **57**:289-300. http://www.jstor.org/stable/2346101
